# Informing One Health Anthrax Surveillance and Vaccination Strategy from Spatial Analysis of Anthrax in Humans and Livestock in Ha Giang Province, Vietnam (1999–2020)

**DOI:** 10.4269/ajtmh.22-0384

**Published:** 2023-01-23

**Authors:** Tan Luong, Tat Thang Nguyen, Van Binh Trinh, Morgan A. Walker, Thi Thu Ha Hoang, Quang Thai Pham, Thi Mai Hung Tran, Van Khang Pham, Van Long Nguyen, Thanh Long Pham, Jason K. Blackburn

**Affiliations:** ^1^ Spatial Epidemiology and Ecology Research Laboratory, Department of Geography, University of Florida, Gainesville, Florida;; ^2^ National Institute of Hygiene and Epidemiology, Hanoi, Vietnam;; ^3^ Emerging Pathogens Institute, University of Florida, Gainesville, Florida;; ^4^ Ha Giang Provincial Center for Disease Control, Ha Giang City, Vietnam;; ^5^ Ha Giang Provincial Sub-Department of Husbandry and Animal Health, Ha Giang City, Vietnam;; ^6^ School of Preventive Medicine and Public Health, Hanoi Medical University, Hanoi, Vietnam;; ^7^ Department of Animal Health, Ministry of Agriculture and Rural Development, Hanoi, Vietnam

## Abstract

Anthrax, caused by *Bacillus anthracis,* has a nearly global distribution but is understudied in Southeast Asia, including Vietnam. Here, we used historical data from 1999 to 2020 in Ha Giang, a province in northern Vietnam. The objectives were to describe the spatiotemporal patterns and epidemiology of human and livestock anthrax in the province and compare livestock vaccine coverage with human and livestock anthrax incidence. Annual incidence rates (per 10,000) for humans, buffalo/cattle, and goats were used to explore anthrax patterns and for comparison with livestock annual vaccine variations. A data subset describes anthrax epidemiology in humans by gender, age, source of infection, type of anthrax, admission site, and season. Zonal statistics and SaTScan were used to identify spatial and space-time clusters of human anthrax. SaTScan revealed space-time clusters in 1999, 2004, and 2007–2008 in the province, including in the northeastern, eastern, and western areas. Most human anthrax was reported between July and October. Most patients were male, aged 15–59 years, who had handled sick animals and/or consumed contaminated meat. High case-fatality rates were reported with gastrointestinal or respiratory cases. Our data suggest that vaccination in buffalo and cattle reduces the disease burden in humans and vaccinated animals but does not reduce the incidence in unvaccinated animals (goats). This study identified spatial areas of high risk for anthrax and can inform One Health surveillance and livestock vaccination planning in contextual settings similar to Ha Giang province.

## INTRODUCTION

Anthrax is a severe and significant zoonosis with potential for a high case-fatality rate (CFR) if not treated in a timely and proper manner.^1^ Anthrax is reported nearly worldwide, with varying intensity and seasonality across its range.^2^^,^^3^ The causative agent, *Bacillus anthracis*, is a spore-forming, gram-positive bacterium with an environmental reservoir. Spores can persist for long periods (years or longer)^4^ under certain conditions, leading to recurrent outbreaks. Anthrax is primarily found in herbivorous wildlife and domestic livestock, with spillover in humans with exposure through handling of sick animals and consumption of contaminated animal products.^5^^,^^6^ There are four main types of anthrax in humans, which are classified by the route of infection: cutaneous (most common), gastrointestinal, respiratory, and (more recently) injectional anthrax.^5^^,^^7^ The three latter types are less frequently reported but have a dramatically high CFR of 25–60%.^1^^,^^8^ Sufficient anthrax vaccine coverage in domestic animals effectively reduces disease incidence in both humans and livestock.^9^

In Asia, anthrax has a broad geographic range, as cases have occurred in several countries, including China, Mongolia, India, Iran, and countries of the former Soviet Union, including Kazakhstan, Kyrgyzstan, and Uzbekistan.^2^^,^^10^ Although current evidence confirms anthrax in neighboring countries, there are gaps in knowledge about zoonoses, including anthrax, in Southeast Asian countries, especially in the Indochina region.^11^

Vietnam is in the tropical latitudes with diverse climatic characteristics ranging from temperate in the northern mountain areas to tropical in the south. Vietnam shares land borders with China, Laos, and Cambodia. Among these, China has reported an average of ∼2,000 cases of human anthrax annually from 1955 to 2014.^10^ Analysis of these cases confirms disease occurrence in China’s border areas, indicating the potential for transborder disease transmission with neighboring countries, particularly where livestock trade is actively operated.^10^^,^^12^ Anthrax has been officially recognized by Vietnamese law since 2007, which mandates the responsibilities of disease control and management to provincial-level authorities.^13^ In 2013, anthrax was prioritized as a zoonotic disease for One Health surveillance and response, involving both human and animal health sectors.^14^ In 2015, human anthrax was made nationally reportable with clearer procedures and guidelines.^15^

In Vietnam, the province of Ha Giang shares a long border with the Yunnan and Guangxi provinces of China, where there is the potential for animal trade between the countries.^16^ Seventy percent of the land in Ha Giang is mountainous, making commuting from the provincial centers to remote areas difficult, and forms subregions with typical agricultural practices and livestock grazing within the province.^16^ Ha Giang province is one of six mountainous northwestern provinces reporting the majority of human anthrax cases in Vietnam.^17^^,^^18^ Livestock anthrax vaccination campaigns were initiated before 2009 and are maintained annually across the province; however, sporadic outbreaks of human anthrax still occur in the province, requiring improved control efforts.

To inform disease surveillance and a focused livestock vaccination campaign, we evaluated the anthrax situation in Ha Giang province from 1999 to 2020 using historical data and reports from human health and animal health sectors. The study objectives were to 1) describe spatiotemporal patterns and the epidemiology of human and livestock anthrax in Ha Giang province and 2) compare variations in livestock vaccine coverage with the incidence of human and livestock anthrax. To address these objectives, we used space-only and space-time scan statistics.

## MATERIALS AND METHODS

### The study setting.

**Figure 1.**
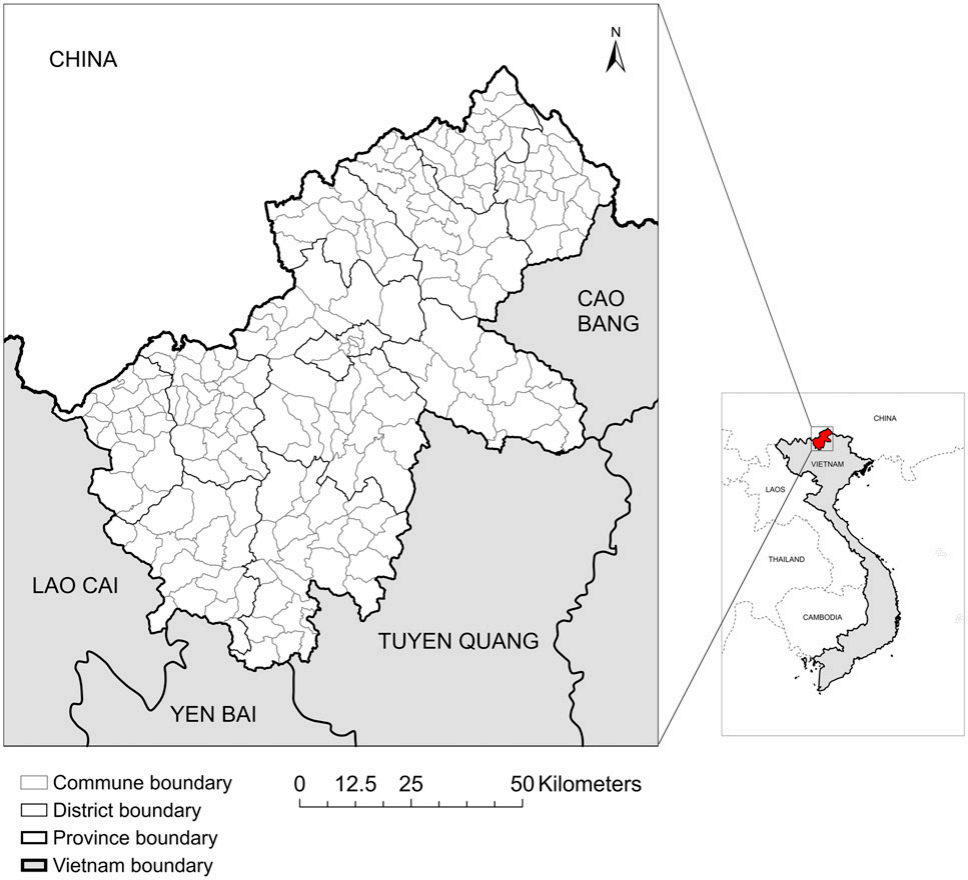
Ha Giang province is located in the north of Vietnam and shares a border with southern China (produced in ArcGIS Pro using shapefiles from www.gadm.org).

Ha Giang province is in the mountainous northwestern region of Vietnam. It shares a northern and northwestern border with China, a northeastern border with Cao Bang province, a southeastern border with Tuyen Quang province, a southern border with Yen Bai province, and a southwestern border with Lao Cai province (Figure 1).^16^ Currently, there are 195 communes (the lowest governmental administration unit of Vietnam) divided across 11 districts in the province. Ha Giang is divided into three subregions based on climatic and topographic features, including the north rocky mountainous subregion (four districts: Quan Ba, Yen Minh, Dong Van, Meo Vac), west soil mountainous subregion (two districts: Hoang Su Phi, Xin Man), and lowland subregion (Ha Giang City and the remaining five surrounding districts). The population of Ha Giang is approximately 870,000 people of 18 ethnicities varying in language, culture, and agricultural practices.^16^

### Data collection and management.

The Ha Giang Provincial Center for Disease Control and Sub-Department of Husbandry and Animal Health (Sub-DAH) were responsible for historical raw data collection corresponding to each human health sector (1999–2020) or animal health sector (2009–2020). The province’s disease reporting system is designed to capture human index cases in healthcare settings (provincial hospitals, district hospitals, and commune health centers) that trigger field outbreak investigations to look for more cases associated with the index cases. In the animal health sector, disease surveillance is conducted through a network of field veterinarians at the district and provincial levels. In this study, anthrax case definitions were consistent with criteria for Vietnam’s national disease surveillance system, which was any case with clinical signs (humans or livestock) and/or symptoms (humans) of anthrax with or without laboratory confirmation (Supplement). Data were aggregated to communes (subdistrict polygons) as the spatial unit analysis for human and livestock anthrax. The commune, district, and provincial polygons were downloaded from GADM data version 3.6 (www.gadm.org).^19^

### Human and livestock population data.

Human population estimates for each commune were derived using a zonal statistics routine on gridded unconstrained individual countries from the United Nations adjusted population counts provided by the WorldPop database for each year from 2000 to 2020 (grid cell resolution is ∼100 m × 100 m at the equator).^20^^,^^21^ The population for each commune in 1999 was estimated from the population in 2000 and an annual growth rate of 1.8%.^22^ The zonal statistic routine was performed in ArcGIS^®^ Pro (Esri, Redlands, CA).^23^ The estimated population was compared with the province-level population from a report of the Ha Giang Province Statistical Office in 2021.^16^ Results of the comparison confirmed that zonal statistics provided a close estimation of the province’s report; the difference between estimations ranged from −0.9% to 1.6% (Supplemental Figure 1).

Livestock here refers to buffalo, cattle, and goats because they accounted for the vast majority of livestock herds in the province. In this study, we separated animals into two groups (buffalo/cattle and goats), as goats made up a large portion of the total herd and were excluded from the anthrax vaccination program in the province. Subsequently, the population and the anthrax incidence for goats were calculated separately from that of buffalo/cattle. The livestock population at the provincial level was provided by a report from either the Ha Giang Department of Statistics in 2021 (2000, 2005) or the Sub-DAH (each year from 2010 to 2020). The gap years in the buffalo and cattle population (1999, 2001–2004, and 2006–2009) were estimated on the basis of the numbers in 2000 and 2005, and an annual growth rate of 3%.^16^ A similar method was used for the goat population but with annual growth rates of 4% (1999, 2001–2004) and 7% (2006–2009).^16^ The annual population of livestock, broken down into buffalo, cattle, and goat components, is presented in Supplemental Figure 2. The population of each livestock species and its contribution (in percentage) was aggregated into district polygons for mapping the distribution of livestock in Ha Giang province.

### Province-level annual incidence of anthrax.

The annual incidence rate at the provincial level was used to analyze temporal patterns of human, buffalo/cattle, and goat anthrax throughout the study period. The incidence for each group was calculated as follows:

$$\mathrm{Incidence}\;\mathrm{rate}\;(\mathrm{per}\;10,000)=\frac{No.\;of\;\mathrm{cases}\;of\;X\;\mathrm{anthrax}\;\mathrm{reported}\;in\;\mathrm{year}\;Y}{\mathrm{Total}\;\mathrm{population}\;of\;X\;of\;\mathrm{the}\;\mathrm{province}\;in\;\mathrm{year}\;Y}\times 10,000$$

where *X* is human, buffalo/cattle, or goat; *Y* ranges from 1999 to 2020.

### Epidemiological description of anthrax in Ha Giang province.

A choropleth map was used to visualize spatial overlap between human anthrax-infected cases, human anthrax deaths, and livestock anthrax cases in ArcGIS Pro. The number of anthrax cases in humans and livestock (buffalo/cattle and goat separately) in each district of Ha Giang province and the annual incidence of human, buffalo/cattle, and goat anthrax at the provincial level were graphed to illustrate the temporal trend of each subject and to compare temporal overlap in cases among the three populations. The number of human cases by type of anthrax and CFR was also graphed. Epidemiological information for human anthrax, including gender, age, self-reported source of infection, type of anthrax, admission/treatment site, month of infection, species of sick/dead animal to which the patient was exposed, and laboratory test results, was available for a subset of cases reported from 2004 to 2020.

### Space-only and space-time cluster analysis by SaTScan statistics.

We were interested in detecting space-only clusters and space-time clusters for the full-time period 1999 to 2020. Space-only and space-time cluster analyses were performed using SaTScan version 9.6.^24^ SaTScan uses a series of search circles of varying diameters, with maximum diameter defined by a proportion of the population at risk (in a Poisson model) to detect spatial clusters across a study area (for space-only and space-time statistics) and a series of cylinders of varying heights to detect temporal clusters throughout the study period (for space-time cluster analysis).^25^ Using the Poisson model, the number of cases in each commune polygon is generated by an inhomogeneous Poisson process considering the null hypothesis that the expected number of cases in each polygon is proportional to its population size and that no covariation exists between the polygons. The Poisson model requires data on number of cases and population of each polygon.^24^ In this study, the Poisson space-only and space-time scan statistics were conducted with year as the time step in the space-time procedure (1999–2020). We evaluated three maximum diameters of search circles at 15%, 25%, and 50% of the population at risk. In each experiment, we set no geographical overlap among clusters and used the longitude and latitude (extracted with the geometry tools in ArcGIS Pro) of the centroid for each commune as the center for each search circle. SaTScan clusters were determined at *P* ≤ 0.05 level of significance. The RR was calculated based on the observed and expected values and the likelihood function of assumed distribution. The likelihood ratio test was used to determine the primary cluster (the value where the likelihood function was maximized) and secondary clusters with lower likelihood function values. For each identified cluster, its center was the commune in which the centroid had been used for determining the center of the search radii for the circles described above, and cluster members were the communes included in the corresponding search circle. Results were aggregated to the communes for mapping and dates of cluster persistence identified.

### Livestock anthrax vaccine coverage in buffalo/cattle.

Anthrax vaccination data in Ha Giang were available for analysis from 2009 to 2020. The total number of anthrax vaccine doses administered annually was provided by the Sub-DAH. In Ha Giang, only buffalo and cattle were vaccinated, one dose per individual per year in accordance with Vietnam’s national guideline.^26^ Thus, annual vaccine coverage was calculated as follows:

$$\mathrm{Annual}\;\mathrm{vaccine}\;\mathrm{coverage}=\frac{\mathrm{Total}\;\mathrm{administered}\;\mathrm{anthrax}\;\mathrm{vaccine}\;\mathrm{doses}}{\mathrm{Total}\;\mathrm{population}\;of\;\mathrm{buffalo}\;\mathrm{and}\;\mathrm{cattle}}\times 100\%$$

### Ethics statement.

This study underwent ethical review by the Institutional Review Board in Bio-medical Research of the National Institute of Hygiene and Epidemiology, Vietnam (IRB-VN01057/IORG 0008555; Project IRB certificate No. NIHE IRB-03/2020) and the University of Florida (IRB202003189).

## RESULTS

### Distribution of buffalo, cattle, and goats in Ha Giang province.

**Figure 2.**
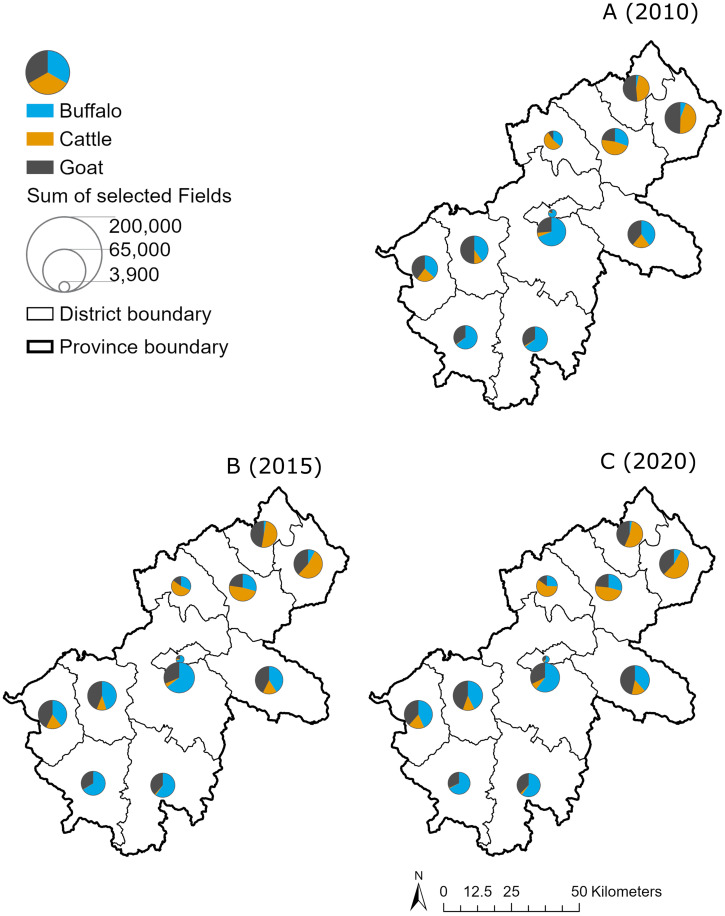
Distribution of livestock by number of livestock heads and species, Ha Giang province, Vietnam. (**A**) 2010. (**B**) 2015. (**C**) 2020.

Figure 2 shows the distribution of three livestock species in 2010, 2015, and 2020. Overall, goats contributed remarkably to the total herd in the whole province, approximately 30%, varying by district. Cattle and goats were increasingly predominant in the northern districts of Ha Giang, whereas buffalo were stable and accounted for a greater proportion of the herd in the south of Ha Giang.

### Annual patterns and geographical overlap of human and livestock anthrax reporting in Ha Giang (1999–2020).

**Figure 3.**
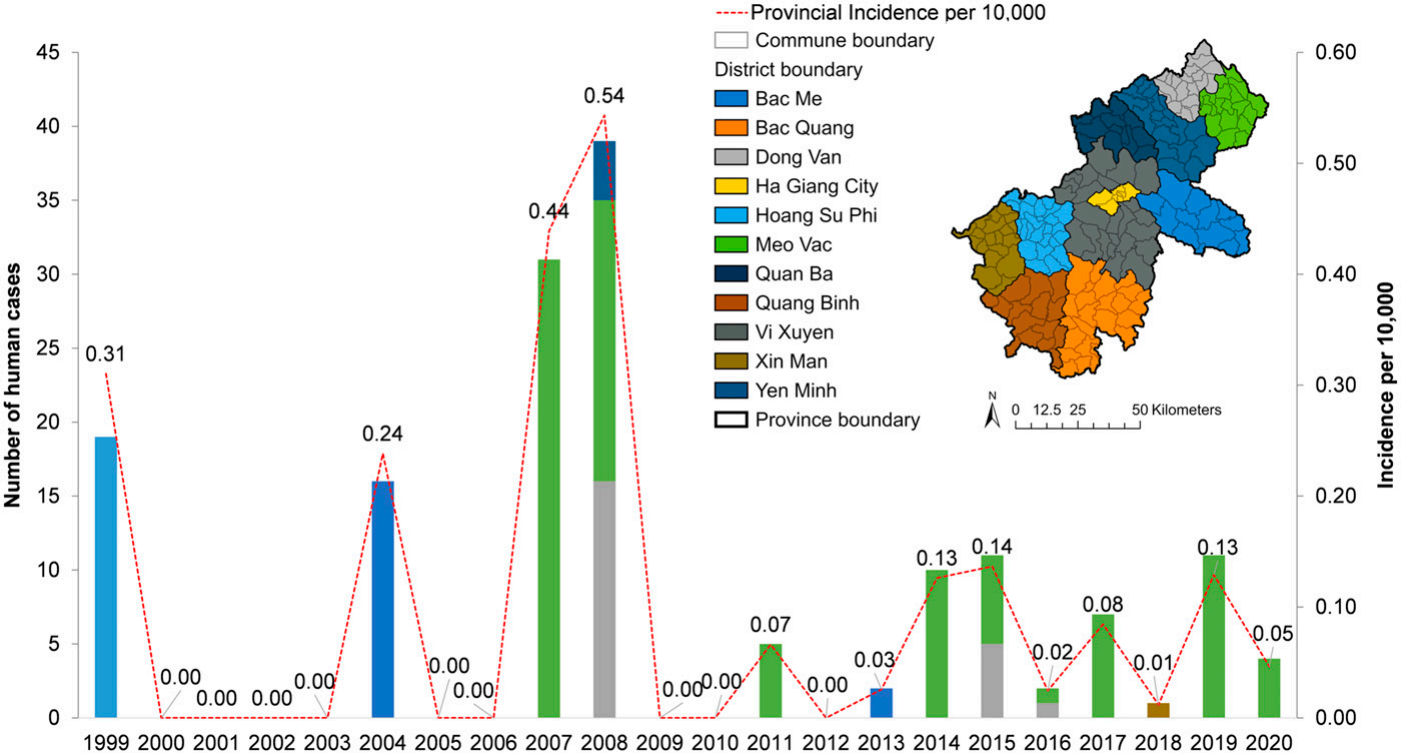
Annual patterns of anthrax by number of cases at the district level and the incidence per 10,000 at the provincial level for humans in Ha Giang province, Vietnam, 1999–2020.

**Figure 4.**
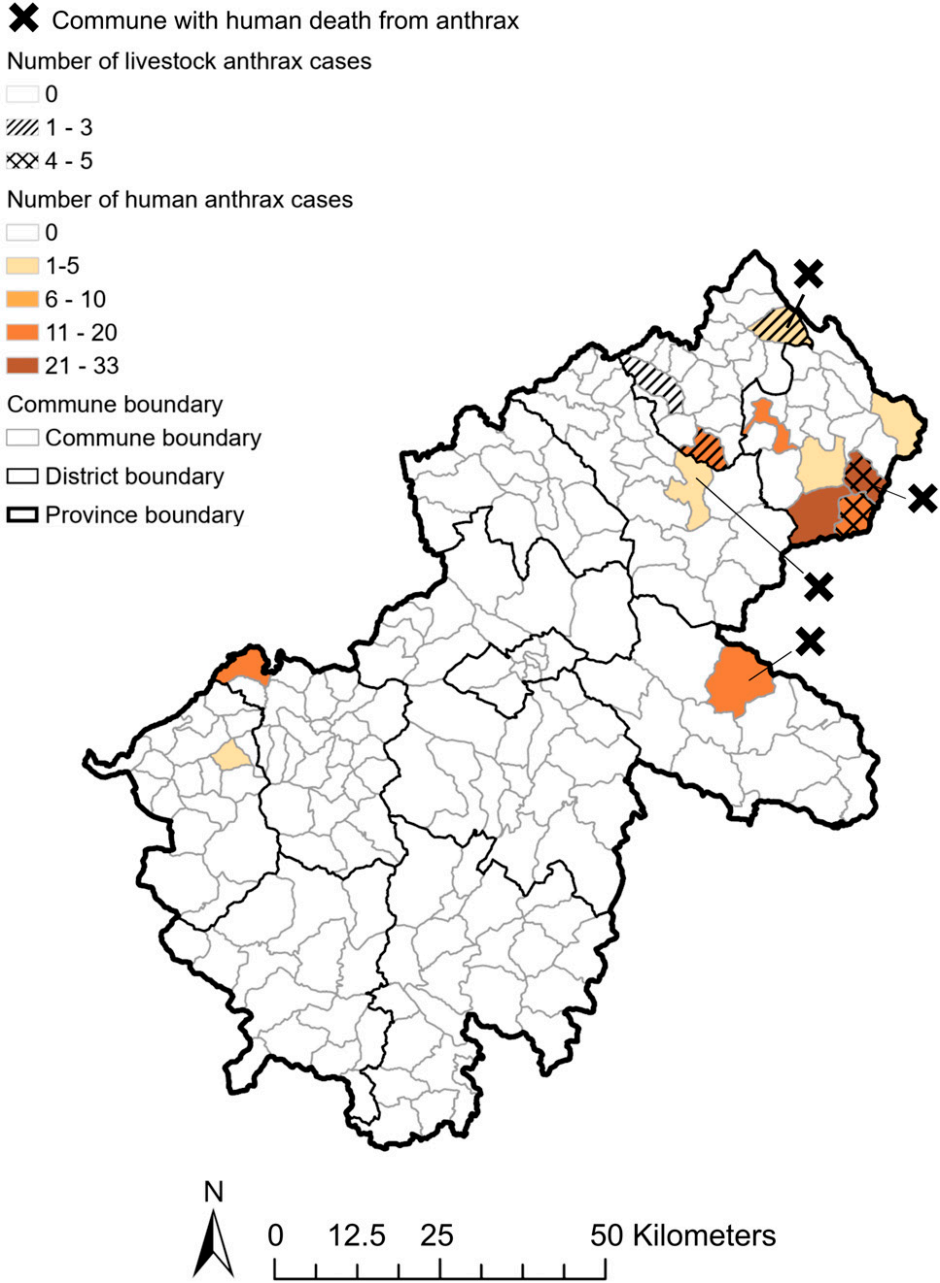
Spatial overlaps of anthrax in humans and livestock, Ha Giang province, Vietnam, 1999–2020.

In total, 158 human cases and 14 livestock cases (nine buffalo/cattle, five goats) were reported from 1999 to 2020 in Ha Giang province. Figure 3 shows that four large outbreaks of human anthrax occurred sporadically between 1999 and 2010, with the highest annual incidence reported in 2008 (0.54 case per 10,000 population). Afterward, small outbreaks occurred more frequently, with lower annual incidence around 0.1 case per 10,000 population. Human anthrax persisted in remote districts such as Meo Vac and Dong Van until 2020. Different patterns were seen for the disease in livestock (Supplemental Figure 3). Livestock anthrax was not recorded in the database before 2009, when human anthrax surged. In 2010, an outbreak in buffalo and cattle occurred, whereas no human cases were reported, resulting in the highest annual incidence of this livestock group (0.19 case per 10,000 buffalo/cattle). In 2014, goats had the highest incidence (0.28 case per 10,000). After 2014, a few cases of anthrax in either buffalo/cattle or goats were reported sporadically. Most of the cases for both human and livestock anthrax were reported in communes in the northeastern regions, with some cases in the southwest. There was spatial overlap of human and livestock anthrax, with a higher number of cases reported in the same communes for both populations. However, anthrax-related human fatalities did not necessarily occur in communes with a higher number of human and/or livestock anthrax (Figure 4).

### Epidemiological characteristics of human anthrax in Ha Giang (2004–2020).

**Figure 5.**
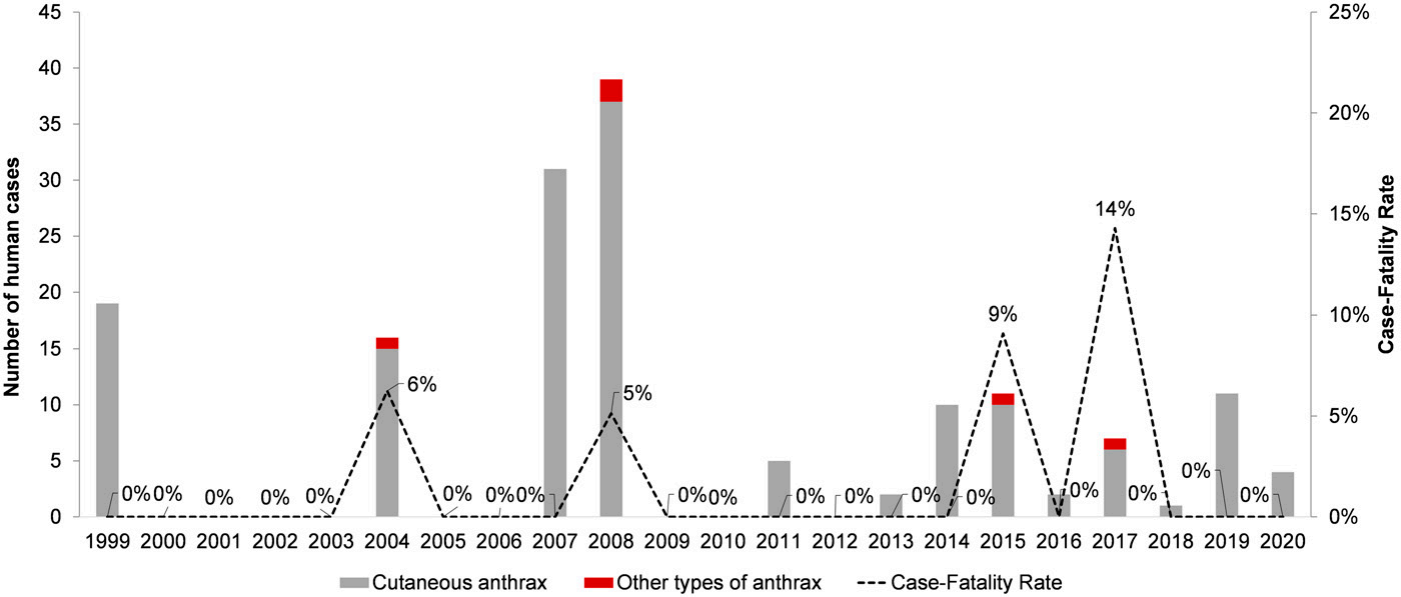
Temporal pattern of human anthrax by types of infection and case-fatality rate, Ha Giang province, Vietnam, 1999–2020.

**Figure 6.**
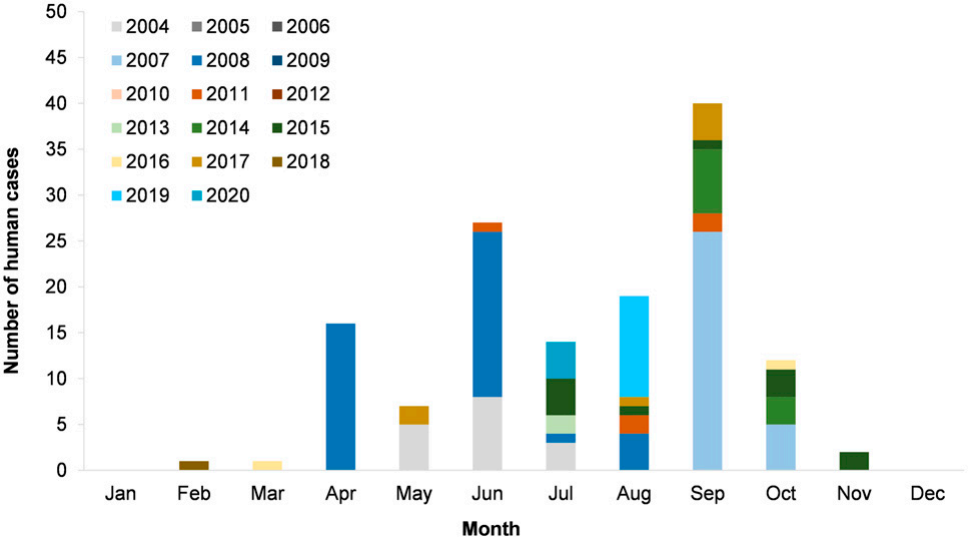
Monthly distribution of human anthrax, Ha Giang province, Vietnam (2004–2020) (*N* = 139).

Among 158 human anthrax cases, 139 cases were reported from 2004 to 2020 with epidemiological data (88% of total cases from 1999 to 2020 and 100% or total cases between 2004 and 2020). Figure 5 shows that the CFR of human anthrax ranged from 0% to 14%, which was higher in the year with types of anthrax other than cutaneous reported. Figure 6 shows the monthly distribution of human anthrax, which indicates three peaks in April, June, and September. However, the peaks in April and June were mainly attributed to a large number of cases in 2007 and 2008. Without the contribution of the large 2007–2008 outbreak, the major season of human anthrax was between July and October, with the peak between August and September.

Table 1 shows some characteristics of the patients. Males were predominant (74.1%). The majority of patients were 15 to 59 years old (71.2%), but a remarkably high percentage was reported for children younger than 15 years (28%). Cutaneous anthrax accounted for 96.4% of all cases. Commune health centers and district hospitals were the main patient admission sites (69.1% and 19.4%, respectively). The majority of cases involved handling of or eating the meat of sick or dead animals prior to the occurrence of anthrax signs and symptoms (84.9%), but unknown sources of infection also accounted for 15.1% of human cases. Information on the type of animals handled and/or eaten by the patients were available for only 46 patients. Among these, 69.6% of patients (32/46) were exposed to sick or dead cattle and horses. Polymerase chain reaction test results were available for 42 patients, of whom 47.6% (20/42) were positive for *B. anthracis*.

**Table 1:** Epidemiological characteristics of human anthrax cases reported from 2004 to 2020, Ha Giang province, Vietnam (*N* = 139)

Characteristic			Frequency	Percentage
Gender	Male		103	74.1
	Female		36	25.9
Age	< 5 years		22	15.8
	5–14 years		17	12.2
	15–24 years		29	20.9
	25–39 years		52	37.4
	40–59 years		18	12.9
	≥ 60 years		1	0.7
Type of anthrax	Cutaneous		134	96.4
	Respiratory		2	1.4
	Gastrointestinal		3	2.2
Site of admission/treatment	At home		15	10.8
	Commune health center		96	69.1
	District hospital		27	19.4
	Provincial hospital		1	0.7
Self-reported source of infection	Slaughter sick/dead animal		8	5.8
	Process meat of sick/dead animal		9	6.5
	Eat meat of sick/dead animal		58	41.7
	Slaughter + eat meat of sick/dead animal		43	30.9
	Unknown		21	15.1
Species of sick/dead animal to which patient was exposed	Available information (46 cases, 33.1%)	Buffalo	6	13.0
		Cattle	16	34.8
		Goat	5	10.9
		Horse	16	34.8
		Pig	3	6.5
	No information (93 cases, 66.9%)			
PCR test result	Available information (42 cases, 30.2%)	Positive	20	47.6
		Negative	22	52.4
	No information (97 cases, 69.8%)			

PCR = polymerase chain reaction.

### Space-only and space-time clusters of human anthrax in Ha Giang (1999–2020).

**Figure 7.**
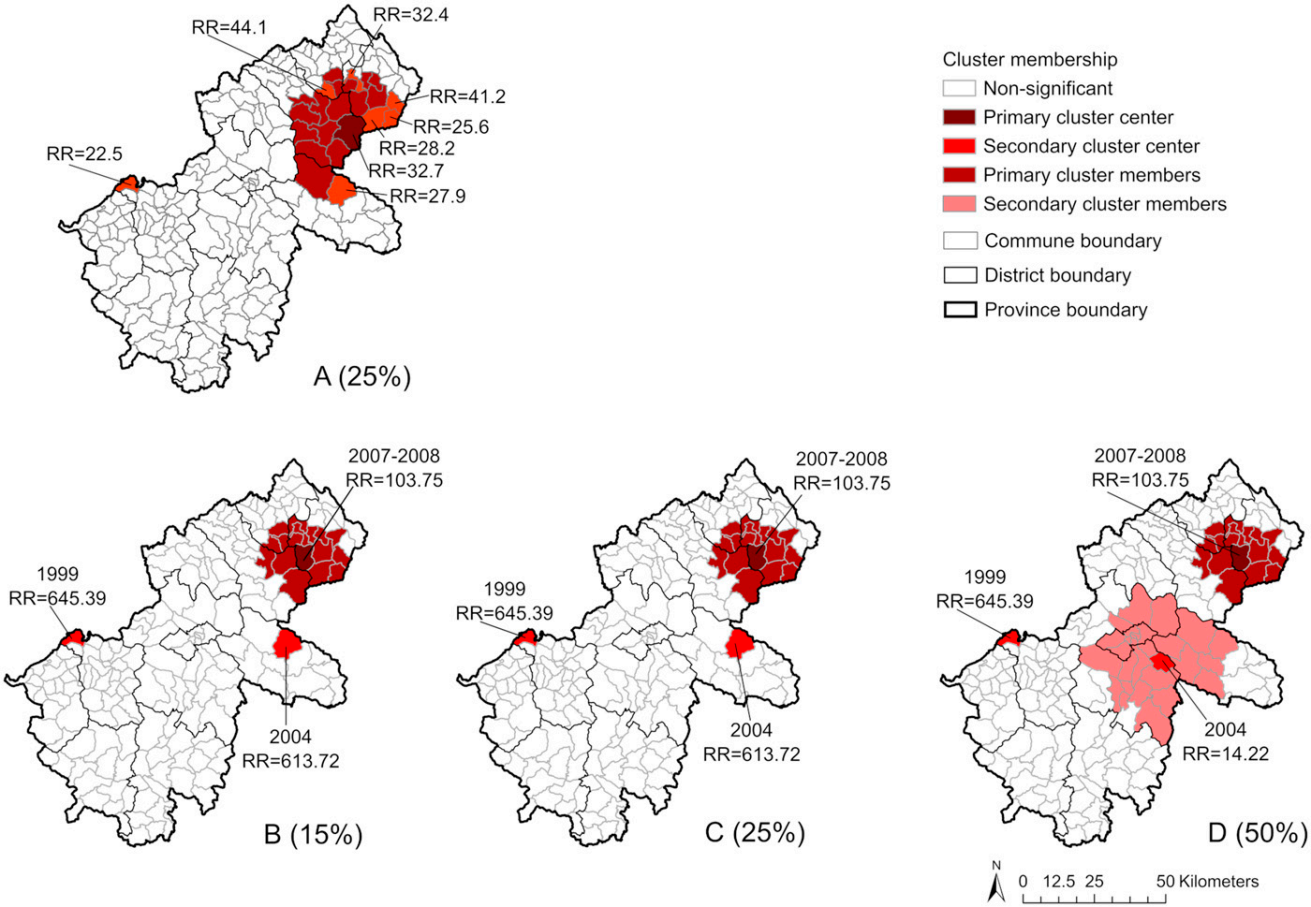
(**A**) Spatial-only clusters (25%; results at 15% and 50% were similar to those at 25%). (**B–D**) Space-time clusters (B, 15%; C, 25%; D, 50%) of human anthrax identified by SaTScan Statistics (Poisson model, 999 permutations, 15%, 25%, 50% of population at risk). The RR indicates the risk of having human anthrax reported for the commune inside of the clusters compared with outside of the clusters.

Space-only and space-time SaTScan statistics performed in three experiments in 15%, 25%, and 50% of the population at risk provided consistent results (Figure 7). All three experiments for space-only scan statistics indicated clusters of human anthrax from northeastern to eastern areas and the western area of the province (Figure 7A presents the results of the spatial scan at 25%). When we considered the time component in SaTScan, the results indicated three space-time clusters in all experiments (Figures 7B–D), in which the primary cluster was identified between 2007 and 2008 in the northeastern communes of the province with a high RR (103.7), meaning that the commune within the cluster is about 104 times more likely to have human anthrax cases reported. Two secondary clusters were identified in the west in 1999 (RR = 645.4) and in the east in 2004 (RR = 613.7 in experiments at 15% and 25% and RR = 14.2 in the experiment at 50%).

### Livestock vaccine coverage in buffalo/cattle and the incidence of anthrax in humans and livestock (1999–2020).

**Figure 8.**
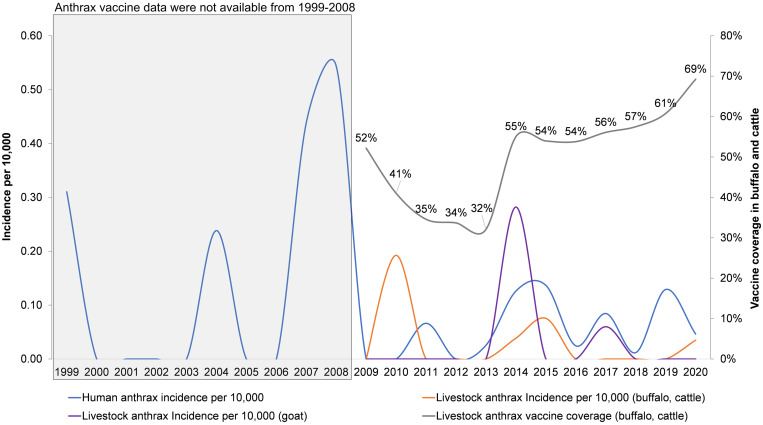
Vaccine coverage in buffalo and cattle and the incidence (per 10,000) of anthrax in humans and livestock, Ha Giang province, Vietnam, 1999–2020.

Figure 8 presents the annual anthrax vaccine coverage in buffalo/cattle from 2009 to 2020 and annual patterns of anthrax in humans (1999–2020), buffalo/cattle (2009–2020), and goats (2009–2020). Vaccine coverage was maintained at 50% or higher of the total herd of buffalo/cattle, which sharply declined to 32–35% between 2011 and 2013. The vaccine coverage drop was followed by an increased incidence in humans, buffalo/cattle in 2014–2015, and goats in 2014, though the vaccination program did not cover this livestock group.

## DISCUSSION

The current study highlights some important results regarding the epidemiological characteristics and patterns of anthrax in humans and livestock from 1999 to 2020 in Ha Giang province, Vietnam. Anthrax vaccine coverage in buffalo/cattle and anthrax distribution in three livestock species were also obtained.

Human anthrax rates were highest early in the study period and became sporadic, with fewer cases reported each year, in the latter half of the study period. Notably, the trend did not flatten during the study, implying the potential occurrence of a larger outbreak if control measures are inconsistent. This is especially true in communes where human and livestock anthrax overlaps and in communes where human population density is increasing. This is an important finding that supports the need to strengthen One Health surveillance in high-risk areas. We demonstrated that when vaccine coverage in buffalo/cattle dropped to 32% in 2013, disease outbreaks in humans, buffalo/cattle, and goats followed in 2014 and 2015. Similar results were reported in the Republic of Georgia, with a significant increase in human cutaneous anthrax after the national compulsory livestock vaccination program ended.^9^ Further, a study in Azerbaijan documented a decrease in human anthrax when livestock vaccination campaigns were reestablished after independence from the former Soviet Union.^9^

Epidemiological data on human anthrax in Ha Giang revealed that most patients were male, were aged 15–59 years, and were involved in slaughtering and/or consuming the meat of sick or dead animals. These results are similar to those of studies conducted in Uganda, Bangladesh, Kazakhstan, and Zimbabwe.^6^^,^^27^^–^^29^ However, further laboratory work and field investigations are needed to uncover the unknown sources of infection in 15.1% of patients and the reasons for an exceptionally high percentage of affected children younger than 15 years (28.0%). Studies from other countries have indicated a lower percentage of patients younger than 15 years.^30^^,^^31^

The number of reported human cases was remarkably higher than the number of reported livestock cases, which raises two issues. First, meat-sharing practices could be common in the study area, where one sick animal may be handled and shared by many people in the neighborhood, resulting in multiple human cases from one infected animal.^18^ Second, livestock anthrax could be underreported in Ha Giang province because of lack of awareness about anthrax and its health and economic impacts among livestock owners, which may be a barrier for anthrax surveillance.^32^ These two issues are particularly relevant to buffalo and cattle in Vietnam, where the animals represent significant, valuable household assets that are important in crop farming practices and could be lost if anthrax is reported.^18^

Nearly all human cases were cutaneous anthrax (96.4%). The other types of anthrax, such as gastrointestinal or respiratory anthrax, were rare but did occur among fatal cases, resulting in relatively high CFRs (5–14%). The CFR is high compared with other studies that reported a CFR of about 1% among cutaneous anthrax patients with proper antibiotic treatments, but lower than the CFR in studies that reported the cutaneous type together with other types of anthrax (i.e., gastrointestinal anthrax).^1^ Commune health centers and district hospitals remained the facilities where most patients (88.5%) sought health services. Nonetheless, we should be cautious when interpreting data reported by limited-resource health facility–based surveillance. In addition to likely underreporting, there is a high likelihood that patients sought treatment from other sources, such as self-treatment, traditional medicine men, and local private pharmacies. This is of particular concern when outbreaks were contained in small groups of ethnic minorities with limited access to health care due to difficult travel conditions in remote areas of the province. A study conducted in 2008 in Vietnam showed that ∼31.0% of poor people relied on self-treatment (i.e., using medicines available at home or buying medicine from drug sellers without a medical examination) and ∼4.2% sought traditional healers.^33^ These scenarios are plausible in the context of remote areas in Ha Giang province. Future studies should address the association between travel distance from the household to human and animal healthcare settings and/or pharmacies and the reported incidence of anthrax in humans and livestock.

From available data, the monthly distribution of human anthrax indicates clear seasonal patterns in Ha Giang province. This is important, as the livestock vaccine should be administered annually ahead of anticipated cases. Such data inform vaccine implementation strategies to provide sufficient vaccine coverage in livestock. Results suggest that the main season of anthrax in Ha Giang province is from July to October, which roughly overlaps the beginning of the rainy season in northern mountainous regions of Vietnam (from June to September).^34^ Studies in Zambia and Kenya indicate an opposite situation in which anthrax outbreaks occurred in the dry season.^35^ However, it was posited that people and livestock in Zambia are exposed to *B. anthracis* in floodplain regions only available or accessible in the dry season.^36^ In Ha Giang province, the mountainous topography and climate create a distinct landscape where pastures are available for livestock grazing in the rainy season and limited in the dry season. A report on livestock production in Vietnam shows that livestock owners in northern regions tend to have their livestock graze in pastural lands at the beginning of the rainy season until the winter comes.^37^ Current literature indicates that the intensity of livestock grazing at the beginning of the rainy season may have plausibly increased livestock exposure to the pathogen in high-risk pastural lands where animal anthrax cases were reported.^5^ Therefore, we suggest the animal health sector consider targeting vaccination campaigns for livestock in April and May, at least 1 month prior to July. This should allow enough time for animals to produce sufficient protective antibody levels.^38^ The seasonality and geographical overlap of human and livestock anthrax in Ha Giang province stress the importance of effective control measures involving the three aspects of the One Health framework, including humans, animals, and the environment. However, more discussion regarding the role of the environmental health aspects of anthrax in disease prevention and control measures is required, as such information is currently limited in the literature.^39^

This spatial and spatiotemporal analysis of human anthrax identified significant and persistent spatial clustering of human anthrax in three major areas: the northeastern region (the largest and most persistent hotspot, with the most recent cluster from 2007 to 2008), the western region, and the eastern region. These high-risk areas also overlapped with the communes where livestock anthrax was reported. These results, together with the seasonal distribution of anthrax described above, are important for targeting surveillance and livestock vaccination programs.

With the available data for anthrax in livestock and vaccine coverage from 2009 to 2020, we defined generally decreasing trends for anthrax incidence in humans and buffalo/cattle, but a stable and slightly increasing trend of anthrax incidence among goats. Studies in other countries indicate the effectiveness of adequate livestock vaccine in reducing the incidence in both humans and livestock.^9^^,^^40^ In the case of Ha Giang province, higher proportions of vaccination reduced anthrax in buffalo/cattle and subsequently in humans. Goat anthrax is not currently controlled with vaccine in Vietnam. The exclusion of goats from anthrax vaccination programs is explained by several reasons. First, some of the available vaccines in the Vietnamese market are not produced or labeled for use in goats, though globally the vaccine is administered in goats.^41^^,^^42^ Second, goats are typically raised for meat production, with a short turnover period (7–8 months), which is difficult to align with vaccine campaigns, particularly when the current vaccination program is implemented in specific months (i.e., from July to August) and often only once per year. Third, goats and cattle are capable of thriving in rocky mountainous regions (northern and northeastern communes of the province). These habitats are a barrier to providing animal health services, including vaccination and disease surveillance. Additionally, anthrax in small ruminants such as goats and sheep is often underreported, though evidence of anthrax in goats was reported in the early 1980s in Nigeria.^43^ A study in Kenya indicates that the ratio of cattle to small ruminants in anthrax reports has been high (10–20 cattle to one small ruminant).^35^ Further work is needed to measure the true pattern of anthrax in goats and its role in the persistence of the disease on the landscape, as well as other factors contributing to vaccination exclusion for goats.

Despite the contribution of this study to identifying areas of higher risk of anthrax and the persistence of the disease in Ha Giang province, there are limitations related to the unavailability of data on livestock anthrax and vaccination before 2010. If data were equally available for both humans and livestock, they could provide a clearer picture of anthrax in livestock and consolidate the results in high-risk areas. Collaborative reporting of anthrax was improved after the establishment of One Health Circular 16 in 2013. Collaborative surveillance provides more complete data than was available prior to the introduction of the circular. This is due to improved data sharing and One Health field investigations between the human and animal health sectors. However, the amount of data here was still biased toward human anthrax. Another limitation is that some human cases were included without laboratory confirmation. However, given the unique clinical presentation of anthrax (particularly cutaneous anthrax) after exposure (handling and consuming of sick animals), we were confident that clinicians correctly identified disease. Nevertheless, we cannot rule out other *Bacillus* spp. causing disease, such as certain strains of *Bacillus cereus* with lethal factor (though reports of these remain limited), including a recent strain isolated from a kangaroo in China.^44^^,^^45^ This limitation is not uncommon in other anthrax studies when the cases were reported late, hindering timely sample collection and pathogen detection.^46^^,^^47^

## CONCLUSION

Here, we provide the spatial and spatiotemporal epidemiology of anthrax in humans and livestock in Ha Giang province, northern Vietnam. These findings directly inform One Health surveillance and the response of provincial authorities in better controlling anthrax. Our study suggests that targeted vaccination at high coverage in buffalo and cattle helped reduce the burden of anthrax in both humans and vaccinated animals, but this should be sustained as cases increase after vaccine declines. Additionally, more work is needed to determine the role of unvaccinated livestock (i.e., goats) in the reoccurrence of anthrax in the province. We have employed spatiotemporal analysis to identify high-risk areas for the occurrence and persistence of anthrax, an approach that can be used in settings similar to Ha Giang province, both within and beyond Vietnam.

## Supplemental files


Supplemental materials
