# Supplementary material for: Informing One Health Anthrax Surveillance and Vaccination Strategy from Spatial Analysis of Anthrax in Humans and Livestock in Ha Giang Province, Vietnam (1999–2020)
Source: Am J Trop Med Hyg. 2023 Jan 23;108(3):492–502. doi: 10.4269/ajtmh.22-0384 (PMC9978550; doi:10.4269/ajtmh.22-0384)
Supplement: Supplementary file 1 [file tpmd220384.SD1.pdf]

SUPPLEMENT

Informing One Health Anthrax Surveillance and Vaccination Strategy from Spatial Analysis of Anthrax in Humans and Livestock in Ha Giang Province, Vietnam (1999-2020)

Luong Minh Tan[1,2,5†], Nguyen Tat Thang[3†], Trinh Van Binh[4], Morgan A. Walker[1,2], Hoang Thi Thu Ha[5], Pham Quang Thai[5,6], Tran Thi Mai Hung[5], Pham Van Khang[5], Nguyen Van Long[7], Pham Thanh Long[7], Jason K. Blackburn[1,2*]

1. Clinical signs and symptoms for surveillance of anthrax in humans and livestock

Human anthrax case definition (Decision number 5703/QD-BYT issued by Vietnam Ministry of Health, dated December 20, 2017):

- Suspected/clinical case is a person who exposes to animal, animal product that is suspected of animal anthrax or living in endemic area. The acute onset signs and symptoms fall into one of the following categories:
  - Cutaneous anthrax: itchy in the infection site, then it forms small blisters, painless black eschar, normally seen around arm, hand, around the patient's mouth and knee.
  - Inhalation anthrax: pneumonia-like symptoms but rapid progress to hard breathing and septic shock.
  - Gastrointestinal anthrax: remarkable abdominal pain, fever, and septic shock.
  - Meningitis anthrax: acute onset, seizure, lost consciousness, and other signs and symptoms of meningitis infection.
- Confirmed case is a suspected/clinical case with one of the following confirmatory tests: bacterium identification by culture, or typical genetic material by molecular biological techniques (PCR).

Livestock anthrax case definition (Circular number 07/2016/TT-BNNPTNT issued by Vietnam Ministry of Agriculture and Rural Development, dated May 31, 2016):

The main signs of anthrax in livestock include tongue exposure, abdominal distention, body fluid containing dark and non-clotting blood run out from mouth, nose, anus, and genital parts. Other signs and symptoms are fever (40°C-42.5^{o}C), high heartbeat rate, shortened breathing, diarrhea,

reduced milk production, abortion, staggering walk, seizure, red eyes. In some cases, the livestock bumps into a bush and dies suddenly or dies after 1-3 days of the onset. Cutaneous symptoms are swollen areas in neck, chest, rump that become cold later, painless, rotten eschar, sometimes forming dark red eschar with yellow fluid. Incubation period in 3-7 days with some exception of 2 days or up to 2 weeks.

2. Supplemental Figures

The supplement includes three figures (Fig S1-S3), which are used to provide additional information to the main narrative. **Fig S1** shows the human population in Ha Giang province, Vietnam for each year from 1999 to 2020 estimated by Zonal Statistic, compared to the populations provided in the Report of Ha Giang Statistic Office. **Fig S2** illustrates the increase in total livestock population and the contribution of each buffalo, cattle, and goat in the total herd in Ha Giang, Vietnam from 1999 to 2020. **Fig S3** shows the annual patterns of anthrax by number of cases at district level and the incidence per 10,000 at provincial level for buffalo/cattle (A), and goat (B), Ha Giang province, Vietnam, 1999-2020.

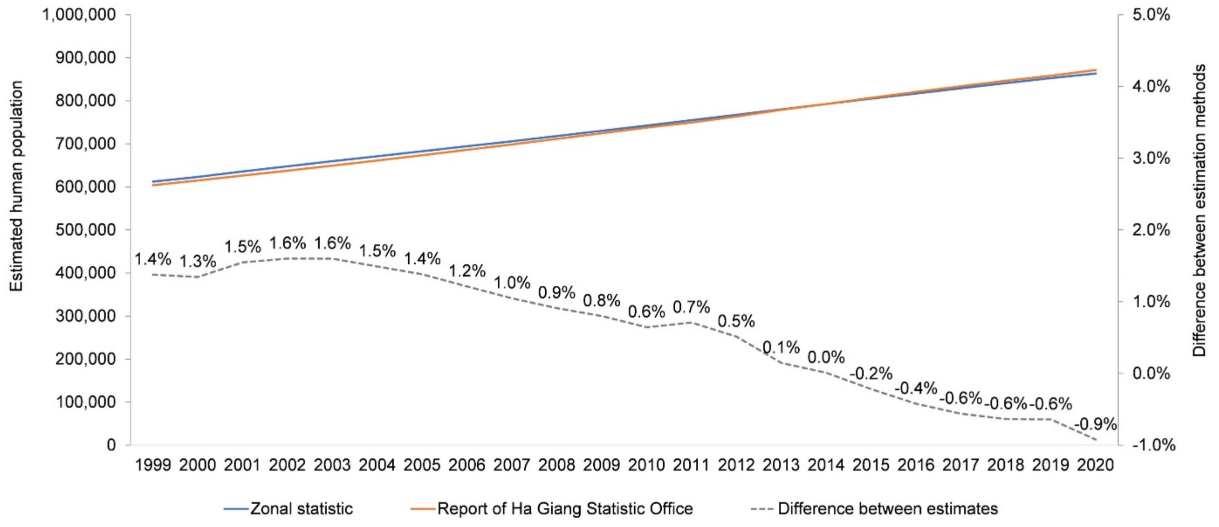

Figure S1. Comparison of population estimation for human using Zonal statistics and the population in the Report of Ha Giang Statistic Office in 2021

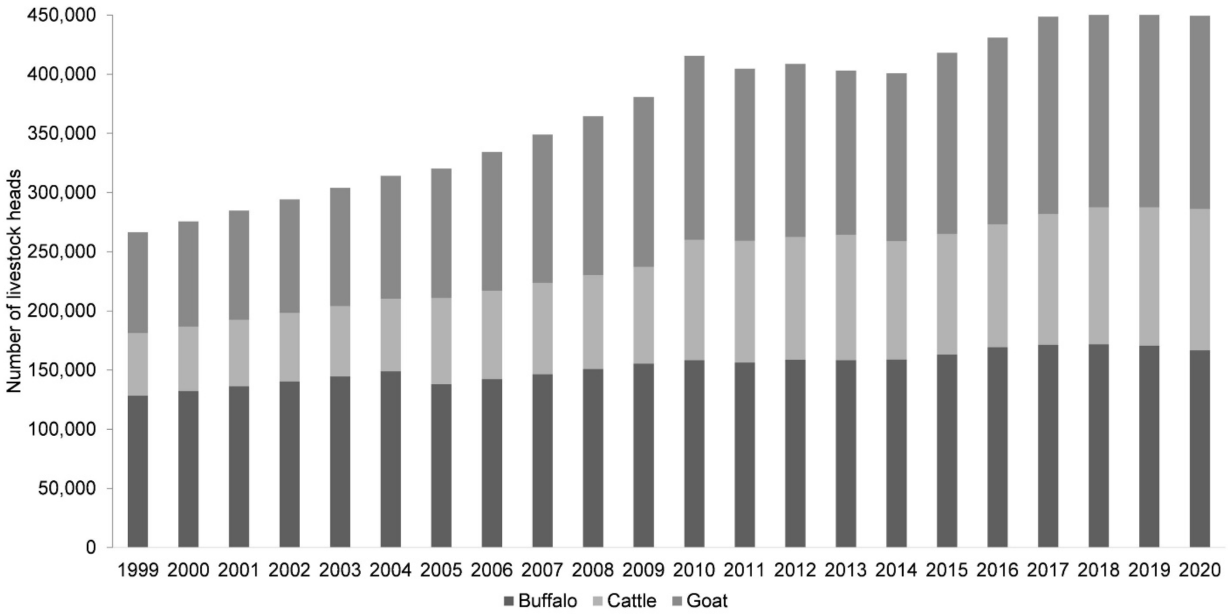

Figure S2. The growth of livestock population and contribution of each buffalo, cattle, and goat in total herd in Ha Giang province, Vietnam, 1999-2020

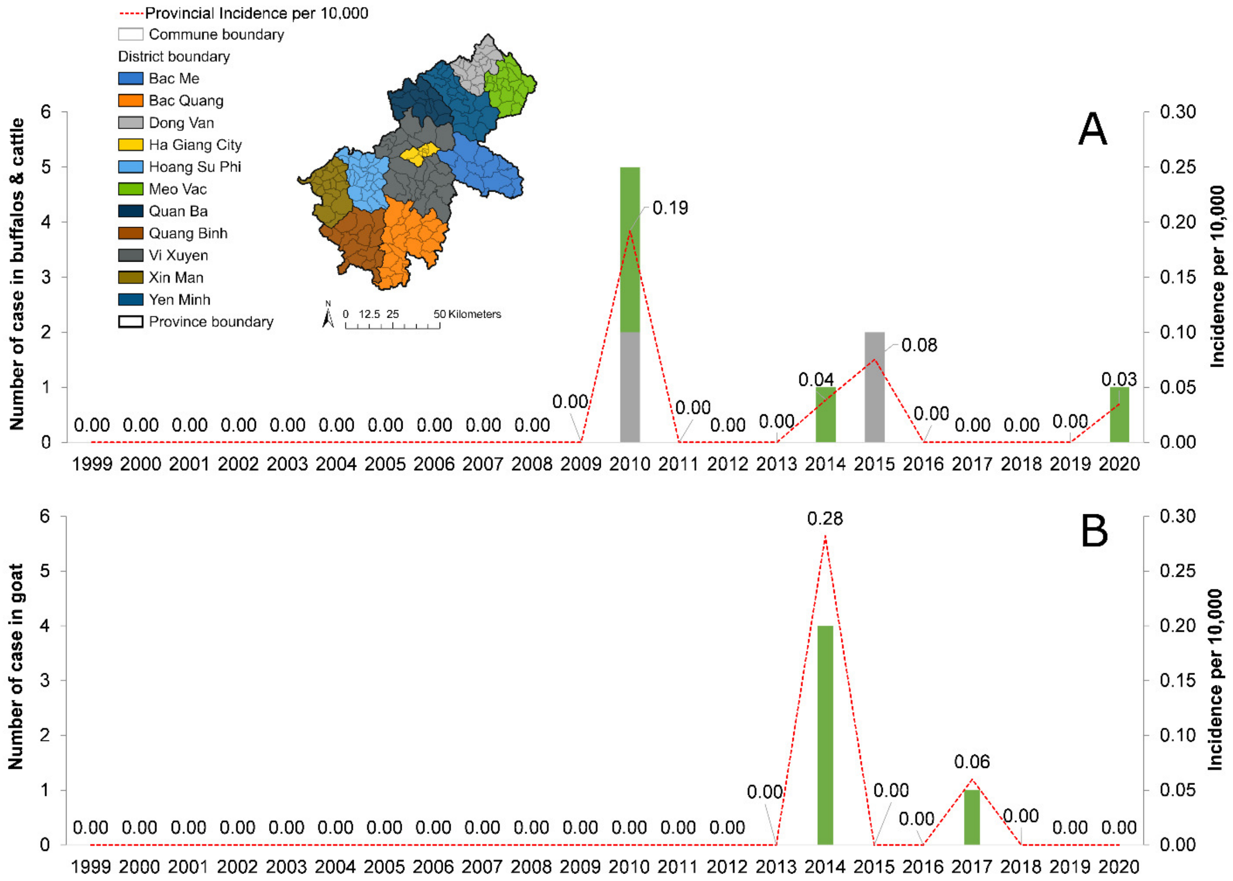

Fig S3. The annual patterns of anthrax by number of cases at district level and the incidence per 10,000 at provincial level for buffalo/cattle (A), and goat (B), Ha Giang province, Vietnam, 1999-2020.
